# Effect of Vaginal Hygiene Module to Attitudes and Behavior of Pathological Vaginal Discharge Prevention Among Female Adolescents in Slemanregency, Yogyakarta, Indonesia 

**Published:** 2017-06

**Authors:** Sumarah Sumarah, Hesty Widyasih

**Affiliations:** Department of Midwifery, Poltekkes Kemenkes Yogyakarta, Daerah Istimewa Yogyakarta, Indonesia

**Keywords:** Vaginal Hygiene Module, Pathological Vaginal Discharge, Prevention, Adolescence, Attitudes, Behavior

## Abstract

**Objective:** To determine the effect of vaginal hygiene module to attitudes and behavior of pathological vaginal discharge prevention in adolescent girls in Sleman Regency, Yogyakarta, Indonesia.

**Materials and methods:** This present study is a quasi experiment with pretest and post-test control group design. A total of 80 female students was selected randomly from two secondary schools at the study site and then distributed equally to control and experimental group. Only participants in the experimental group were given self-learning vaginal hygiene module to maintain vaginal cleanliness. A questionnaire was used as an instrument to measure the attitudes and behavior of vaginal discharge prevention. Paired and independent sample t-tests with significance level (p value) at 0.05 and *Confidence Interval *(CI) of 95%were employed to compare the mean difference.

**Results:** There is a significant difference in the students’ attitude and practice of pathological vaginal discharge prevention between intervention and control group. The students who have been exposed to vaginal hygiene module for six months showed better attitudes and practice in pathological vaginal discharge prevention compared to their counterpart in control group who do not receive any module.

**Conclusion:** The present study implied that vaginal hygiene module can be provided widely for female adolescents at their early puberty. District health officers may work closely with schools’ health promoter to reach the students and create a supportive environment for reproductive health discussion and forum in order to achieve better adolescents’ reproductive health status.

## Introduction

As a result of the past fertility decline, Indonesia is now constituted of large portion of young people aged 10-24 years old (1, 2). The increasing number of young people also followed by an increasing concern since this group of population is vulnerable to sexual and reproductive health problems. Studies found, adolescent females represent a newly menarche group, and have their ownsusceptibility with the influence of reproductive hormones (3). Vulvo-vaginitis is the most common gynecological diseases in prepubertal and adolescent girls (4), suffered by more than 60 percent adolescents worldwide. Vaginal discharge, erythenla, and pruritus were common as the symptoms with *Candida sp., Streptococci sp.* and *Enterococci sp.* as the most frequent pathogen involved (5, 6). 

Vaginitis is commonly found among women at all age, but the prevalence is higher among women at reproductive age (7). Adolescents are prone to vaginitis because there is a lack of labial fat pads and pubic hair, small labia minora, thin vulvar skin and the closeness of the vulva to the anal region. Young adolescents are also low in glycogen, with neutral pH and non-production of cervical mucus and poor local immune system (4, 6). The likelihoods of infection are also elevated by poor hygiene derived from lack of knowledge in reproductive health, especially in genital organs (4, 6). Low absorbed of underwear, panty liner, and pads may increase the yeast infection, especially when it’s used in a long period. In some settings, the practice of inappropriate genital washing such as using inappropriate intimate detergents (6) and vaginal douching have been pointed as the causes of infertility among women and therefore is no longer recommended as methods in genital organ washing (8, 9). 

Moreover, high humidity as the general climate in Indonesia may increase the probability of yeasts infection such as *Candida sp. *It is estimated, yeast infection contributed to 20-30 percent of the cases in vaginitis. *C.albicans* and *C.glabrata* maybe found in women with recent courses of antibiotics, corticosteroid treatments or those who suffered from diabetes. Pregnancy, exogenous estrogens, use of broad spectrum antimicrobials, immunosuppression, and possibly sexual intercourse are also found as the predisposing factors of yeast infection. The recovery of yeasts in girls during pubertal maturation is also more frequent, generally associated with the presence of vaginal Lactobacilli (6, 10). 

As well as in other regions of Indonesia, the number of female adolescents aged 10-14 in Yogyakarta is estimated to increase every year, from 126,000 in 2014 to 132,000 in 2020 (11). Sleman as the most populated district also risked to higher burden of health problem with the increasing number of adolescents, especially when discussion about reproductive health and sexuality remains taboo in Javanese context. Studies found, lack of reproductive health knowledge has indeed brought young people into a greater risk of unwanted pregnancy and sexually transmitted disease, including HIV and AIDS in their later life (12, 13). 

Many female adolescents are having poor reproductive health practice. Although they acknowledge vaginal discharge as the vaginitis symptoms, many do not understand the cause and prevention. Preliminary study in Sleman State Secondary School revealed that 80 percent of students experiencing vaginal discharge. Of them, 60 percent reporting continued whitish or yellowish discharge of mucus from the vagina during the past three months. This study aims to determine the effect of vaginal hygiene module to attitudes and behavior of pathological vaginal discharge prevention in adolescent girls in Sleman district, Yogyakarta, Indonesia.

## Materials and methods

The present study is a quasi experiment with pretest and post-test control group design. It examined the effect of the provision vaginal hygiene module to the attitudes and behavior of pathological vaginal discharge prevention in adolescents. Following the large number of cases of vaginal discharge among teenagers in Sleman regency Yogyakarta, a total of 80 female students was selected randomly from two secondary schools in the study site. The involved adolescents were then distributed equally to control and experimental group. Only participants in the experimental group were given self-learning vaginal hygiene module to maintain vaginal hygiene. 

A questionnaire was employed as an instrument to measure the attitudes and behavior of vaginal discharge prevention in female teenagers representing by the total score of cognitive, affective, and conative assessment. The pretest was given before the participants obtained the module. The posttest was done 6 months after the first observation. Paired t-test was employed to decide whether the mean score of the observed variables from pre and posttest is significantly different while independent sample t-test was used to find the significant mean difference between control and experimental groups. Statistical analysis was performed with significance level (p value) at 0.05 and *Confidence Interval *(CI) of 95%. 

The present study obtained its approval from Ethical Committee Board of Politeknik Kesehatan Kemenkes Yogyakarta (Yogyakarta Health Polytechnic), Indonesia, with reference number LB.01.01/KE/XVII/097/2016, on April 26th, 2016.

## Results

Generally, most of students from both intervention and control group lived in urban settings although the proportion of respondents in intervention group who lived in rural area slightly lower than the proportion of respondents in control group. Initially, the vaginal hygiene practices of students in intervention group were observed slightly better in washing hands practice, choosing menstrual pad, wearing cotton fabric panties and the selection of daily outfits. At the other hand, students in control group were better in terms of changing panties and changing menstrual pads. After the provision of vaginal hygiene module, more students in intervention group showed better vaginal hygiene practices compared to their control counterpart. As it shown in table 1, the vast majority of respondents in both intervention and control group depicted a good practice in changing menstrual pads, but not in wearing cotton fabric panties and changing panties after exercise. The percentage of students who change panty liners at least 4 times a day significantly increased from 2.5% to 37.5% whilst those who used to wear tight jeans are now wearing loose pants. Students in the control group on the other hands, did not show an improvement in their practices.

The students in the intervention group showed an improved attitudes after the provision of vaginal hygiene module. Initially, only 5% had correct attitudes of vaginal douche products but after the intervention, 45% had positive attitudes as expected. The students also showed better attitudes toward changing panties after exercise, showed by the percentage who agreed was increased from 15% in the beginning to 32.5% after the introduction to vaginal hygiene module. The students who were less likely to change their pads at school, changed their thoughts and believed after the correct information was provided. Although significant attitude changes were observed in terms of changing menstrual pads while at school, it should be noted that the students’ knowledge on the use of soap as vaginal douche was lacking. After intervention, it was only 15% of students in the intervention group and 7.5% of students in the control group understood and had a correct attitude. 

Table 2 presents the mean score (± standard deviation) of attitudes and behavior to vaginal discharge prevention among the participants at the first and second observation. The findings indicate the mean difference of students’ attitude and behavior score at the beginning in both control and experimental group is not significant. The data suggests that at the second observation the scores of students’ attitude and behavior tend to increase. Observed at the control group, the attitude score at the control group slightly increased from 67.18 ± 4.517 to 69.00 ± 4.857, whilst the score of behavior was monitored improved only at 1.15 point. The significant changes of student attitudes and behavior were noticed at the experimental group. The attitude score obviously rose from 66.85 ± 4.023 to 76.12 ± 6.646 (p value < 0.001) whilst the behavior score before and after given vaginal hygiene module was remarkably different with an estimated value of 7.82 ± 4.187 (p value < 0.001).

From table 3, the mean differences of adolescences’ attitudes and behavior to the observed genital discomfort between control and experimental group were notably different (p value < 0.001). The data suggests that the given vaginal hygiene module significantly changes the students’ response to pathological vaginal discharge prevention. The students’ attitudes and behavior were scored better at the experimental group than the control group.

**Table 1:** Percentage of correct practice in vaginal hygiene

**Vaginal hygiene practice (selected questions)**	**Intervention**		**Control**	
	**(%)**			
	**Pretest**	**Posttest**	**Pretest**	**Posttest**
Changing panty liners at least 4 times a day	2.5	37.5	0.0	5.0
Washing hands before washing vagina	55.0	77.5	40.0	57.5
Changing panties at least 2 times a day	60.0	75.0	70.0	62.5
Sprinkle powder to vagina when it feels itchy	72.5	85.0	80.0	80.0
Changing panties after exercise	27.5	42.5	45.0	35.0
Choosing menstrual pads	42.5	72.5	35.0	37.5
Wearing loose pants for daily outfits	12.5	62.5	7.5	17.5
Wearing cotton fabric panties	27.5	55.0	7.5	15.0
Changing menstrual pads	80.0	90.0	87.5	77.5

**Table 2:** The mean score (± standard deviation) of students’ attitudes and behavior of pathological vaginal discharge prevention pretest and posttest in control and experimental groups

**Variables**	**Group**	**Score (mean ± std. deviation)**		**95% CI**	**p** **value**
		**Pretest**	**Posttest**		
Attitude	Control	67.18 ± 4.517	69.00 ± 4.857	-1.825	0,039
	Experimental	66.85 ± 4.023	76.12 ± 6.646	-9.275	0,00
Behavior	Control	50.65 ± 3.317	51.80 ± 3.611	-1.150	0,144
	Experimental	51.15 ± 4.759	58.98 ± 4.252	-7.825	0,00

The score of students’ attitudes and behavior at the experimental group was significantly higher after the students exposed by the module, with a score of 9.28 ± 5.533 and 7.82 ± 4.187, respectively.

**Table 3:** The mean difference (± standard deviation) of students’ attitudes and behavior of pathological vaginal discharge prevention in control and experimental group

**Variabel**	**Group**	**Score**	**p** **value**
Attitude	Control	1.82 ± 5.406	0.00
	Experimental	9.28 ± 5.533	
Behavior	Control	1.15 ± 4.876	0.00
	Experimental	7.82 ± 4.187	

## Discussion

Social construction of Javanese culture has prevented the discussion about reproductive health matters to unmarried youth. About one fourth of young women never talk with anyone about menstruation before they have their first menses, and half of young men never discussed wet dreams before their first wet dream (1, 14, 15). Parents are the last person young people seek for information regarding reproductive health whilst schools only provide limited information on anatomy and physiology of human bodies. In the absence of adequate information, young people mostly access media to fulfil their curiosity regarding their own sexuality. Unfortunately, given the nature of media as double-edged sword in providing information, coupled with limited searching skills, many youngsters are then finally ended up to access unreliable sources of information (16).

Studies found that school is proven as a reliable source of reproductive health information for young people. Adolescents who obtain adequate and accurate information from schools are more likely to have better knowledge, attitude and reproductive health behavior (12, 13). Nevertheless, because providing reproductive health education is often mixed-up with the controversial sex education, school’s curricula mostly only provide limited courses incorporated into Biology or sport science. Whilst adolescents need more space to discuss the changes in their bodies, little is known what schools can accommodate. 

The present study attempts to bridge the gap of information that adolescents needed. Vaginal hygiene module is designed for students as structured education program. The module enables students to independently explore their curiosity and facilitate them to have better understanding of the subjects. The results showed there is a significant difference in the students’ attitude and practice in pathological vaginal discharge prevention between intervention and control group. The students who exposed to vaginal hygiene module for six months showed better attitudes and practice in pathological vaginal discharge prevention compared to their counterpart in control group who do not receive any module. The study implies, knowledge improvement may lead to positive attitudes and better practice, because attitudes gradually change with supportive environment. The module as one of supportive elements has its strength in providing access at the users’ convenience. Adolescent females who reluctant to discuss about their genital organ may access the module anytime and overtime, thus, providing a better understanding of the sensitive issue. 

Theoretically, human behavior is considered as a result of cognitive process, including perception that predicts the likelihood of individuals in engaging the intended behavior. Behaviors (response) are also influenced by information received from the environment (stimulus). Perception is also proceeded by stimulus which contained of information than can be stored and recalled anytime (17, 18). Not only affecting the cognitive aspect, the 6-months continuous exposure from the module also showed the students’ motivation in finding information regarding their own reproductive health care and prevention. Students who have positive attitudes toward pathological vaginal discharge prevention are more likely to have better practice and ultimately will reduce the probability of infection.

It is true that the provision of vaginal hygiene module was able to improve attitudes and practice, shown by the overall score of students from pretest to posttest. However, attention should be addressed to specific behavior where most adolescents had a poor practice such as changing panty liners and wearing tight jeans as daily outfits. In general, the vast majority of students do not understand that they have to change panty liners at least 4 times a day to prevent pathological vaginal discharge. After the intervention, only 37.5% of students changed their behavior. The likelihood of attitudinal changes was also less in terms of changing their daily outfits. The information provided in the module was unable to change the students’ preference for tight jeans. The economical cost of behavior changes perhaps can explain why their attitudes are less likely to change. Changing panty liners four times a day of course will be an additional burden for their parents while changing their existing daily outfits will be costly too. Therefore, health promoter should be very careful in conveying the message especially if it’s related to additional cost of behavior changes. 

Unlike practice that can be observed, attitudes somehow are difficult to be measured. However, attitudinal changes were captured from the posttest results. Although significant attitude changes was observed in terms of changing menstrual pads while at school, it should be noted that the students’ knowledge on the use of soap as vaginal douche was lacking. While knowledge was not assessed in the present analysis, the students’ knowledge was somehow reflected from their attitudes. Misbelieved and misunderstanding on the use of soap as vaginal douche therefore should be the center of attention for the health providers for the future health promotion programs. 

It should also be noted that human behavior itself is a result of individual and environmental forces. The long duration (6 months) of intervention also should rise a precaution of contamination. Since it is not possible to isolate the subject of intervention, the knowledge, attitude and behavioral changes probably are not only the results of the module provision but as their interaction with external factors such as their peers and other media exposures. Therefore, further research should seek a better methods in measuring the net impact of the health education tools to behavior changes. Furthermore, since adolescents are socially active and not possible to be isolated, future research should combine several possible factors such as peers and media to obtain a better estimate of behavioral changes.

***Conclusion:*** There is a significant difference in the students’ attitude and practice of pathological vaginal discharge prevention between intervention and control group. The students who exposed to vaginal hygiene module for six months showed better attitudes and practice in pathological vaginal discharge prevention compared to their counterparts in control group who do not receive any modules. The results implied that vaginal hygiene module can be provided widely for female adolescents at their early puberty. The information provided in the module should address specific behaviors where most adolescents had poor practice such as changing panty liner and wearing loose pants. District health officers should work closely with schools’ health promoter to reach the students and create a supportive environment for reproductive health discussion and forum in order to achieve better adolescents’ reproductive health status.
